# Race Does Not Predict Melanocyte Heterogeneous Responses to Dermal Fibroblast-Derived Mediators

**DOI:** 10.1371/journal.pone.0139135

**Published:** 2015-09-29

**Authors:** Pornthep Sirimahachaiyakul, Ravi F. Sood, Lara A. Muffley, Max Seaton, Cheng-Ta Lin, Liang Qiao, Jeffrey S. Armaly, Anne M. Hocking, Nicole S. Gibran

**Affiliations:** University of Washington Department of Surgery, Seattle, Washington, United States of America; Rutgers University, UNITED STATES

## Abstract

**Introduction:**

Abnormal pigmentation following cutaneous injury causes significant patient distress and represents a barrier to recovery. Wound depth and patient characteristics influence scar pigmentation. However, we know little about the pathophysiology leading to hyperpigmentation in healed shallow wounds and hypopigmentation in deep dermal wound scars. We sought to determine whether dermal fibroblast signaling influences melanocyte responses.

**Methods and Materials:**

Epidermal melanocytes from three Caucasians and three African-Americans were genotyped for single nucleotide polymorphisms (SNPs) across the entire genome. Melanocyte genetic profiles were determined using principal component analysis. We assessed melanocyte phenotype and gene expression in response to dermal fibroblast-conditioned medium and determined potential mesenchymal mediators by proteome profiling the fibroblast-conditioned medium.

**Results:**

Six melanocyte samples demonstrated significant variability in phenotype and gene expression at baseline and in response to fibroblast-conditioned medium. Genetic profiling for SNPs in receptors for 13 identified soluble fibroblast-secreted mediators demonstrated considerable heterogeneity, potentially explaining the variable melanocyte responses to fibroblast-conditioned medium.

**Discussion:**

Our data suggest that melanocytes respond to dermal fibroblast-derived mediators independent of keratinocytes and raise the possibility that mesenchymal-epidermal interactions influence skin pigmentation during cutaneous scarring.

## Introduction

Abnormal pigmentation following cutaneous injury causes significant patient distress regarding body image and exacerbates psychosocial outcomes including low self-esteem and social isolation especially in people of color [1,2]. Wound depth and pre-injury skin color and ethnicity influence late scar pigmentation [3]. Limited understanding of melanocyte responses to injury has stalled development of therapeutic solutions to restoring skin color [4–6].

Epidermal pigmentation after cutaneous injury requires melanocyte proliferation, migration into the wound, melanin production, and pigment transfer to neighboring keratinocytes. Clinical observations in humans and animal models suggest that melanocytes migrate from both the wound edge and epidermal appendages after wound epithelialization [7]. Melanocyte stem cell migration across the epithelialized wound likely depends on melanocortin 1 receptor signaling [8] and inflammatory responses [9–11]. Immune cell depletion in zebra fish reduces melanocyte recruitment to the wound resulting in decreased wound pigmentation [12]; whether this holds in mammalian wounds is less clear. Excessive and prolonged inflammatory responses to cutaneous injury are associated with hypertrophic scars and may lead to abnormal pigmentation [10]. However, the molecular signals responsible for inducing melanocyte responses to injury remain unknown.

Most melanocyte biology studies have involved interactions with keratinocytes [13,14], since the cells reside in the same cutaneous compartment and have cell-cell contact. However, melanocytes interact with fibroblasts [15–17], potentially implicating epidermal-mesenchymal interactions in regulation of cutaneous morphogenesis. Recent evidence supports melanocyte modulation of angiogenesis [18,19], inflammation [20], and fibrosis [10] after injury. Based on the clinical observation that wound depth influences both eventual scar pigmentation [7] and hypertrophic scar formation [21,22], we hypothesized that phenotypic and genomic responses by melanocytes to mesenchymal signaling represent an epidermal-mesenchymal interaction after cutaneous injury.

## Methods and Materials

### Human epidermal melanocytes

Six human neonatal primary epidermal melanocyte isolates were purchased (Life Technologies; Grand Island, NY) having been isolated from single male donor foreskin less than 14 days old. According to company specifications, three donors were Caucasian (C1-3), one of whom was Hispanic (C1), and three were non-Hispanic African American (AA1-3). Cryopreserved cells were thawed and expanded in Medium 254 supplemented with human melanocyte growth supplement-2 and penicillin-streptomycin as recommended by Life Technologies. Cells were used at passages 4–10 for experiments.

### Melanin assay

**Fig 1:**
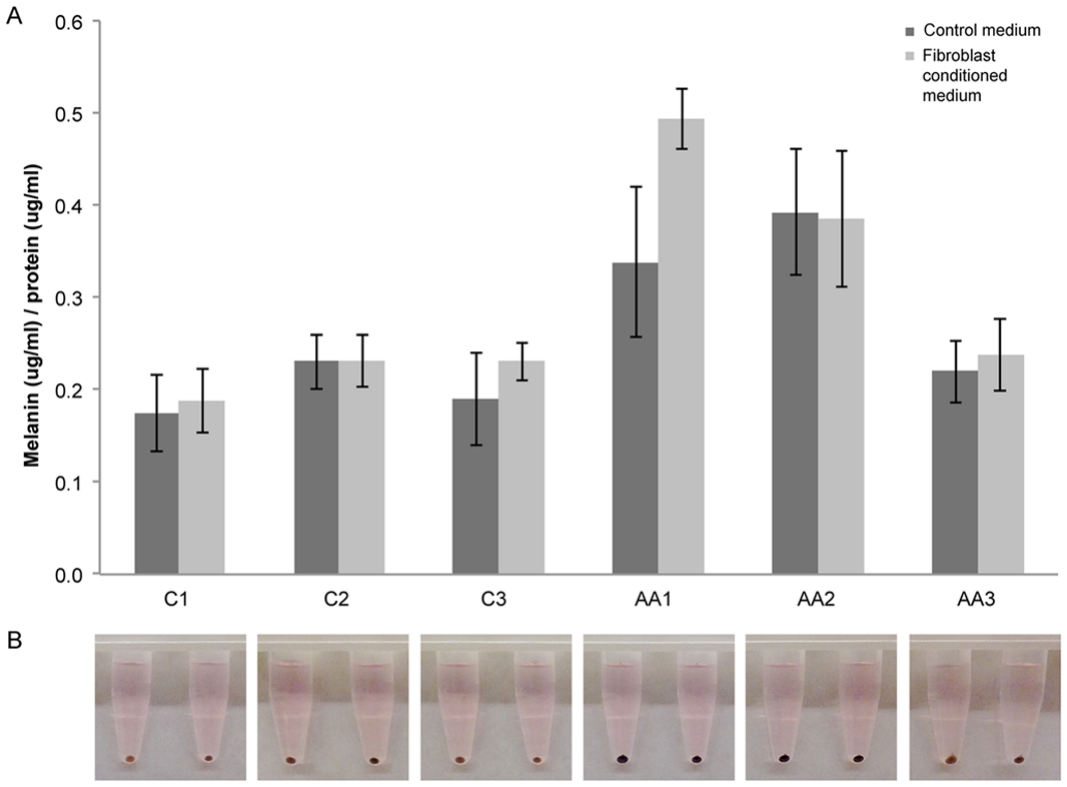
Melanin production is highly variable across melanocyte lineages. a) Melanin production varied significantly between cell lineages at baseline (*p*<0.0001) and in response to fibroblast-conditioned medium (*p* = 0.01); the magnitude of response to fibroblast-conditioned medium varied significantly across samples (*p* = 0.03). Melanin production was not consistent with either reported race or pigmentation type as assigned by Life Technologies. Statistical analysis was based on average values across all six samples rather than individual sample responses to fibroblast-conditioned medium (see Methods). b) Photodocumentation of cell pellets from each of the melanocyte cell lineages demonstrated no dramatic differences in melanin appearance.

We performed melanin assays on each set of cells (Fig 1). Cellular melanin content was determined as described [15]. Melanocytes were cultured in T150 flasks in standard Medium 254. On day -1, medium was switched to Fibroblast-conditioned medium or a 1:1 mix of Medium 106/Medium 254 as the control. After 24 hours, supernatant was collected and adherent cells were trypsinized and counted. One million cells per cell lineage were pelleted (12,000g) and photographed. Pellets were suspended in 300ul of 1N NaOH and incubated for 30 minutes at 100°C. After vortexing, samples were centrifuged at 12,000g for 10 minutes. Supernatant and cell extracts were transferred to microwell plates and melanin concentration was measured at 405nm using a Spectomax^®^ Plus (384) Plate reader (Molecular Devices, Sunnyvale, CA). Intracellular melanin concentration, expressed as μPlus (384), was back calculated based on a standard curve generated from synthetic melanin (Sigma-Aldrich, Saint Louis, MO).

### Single nucleotide polymorphism analysis in melanocytes

Given that pigment phenotype is determined by single nucleotide polymorphisms (SNPs) in genes responsible for pigmentation [23], we genotyped the melanocytes for 542,585 SNPs spanning the entire genome using a HumanCoreExome-12 Bead Chip (Illumina, San Diego, CA) according to manufacturer protocol. In addition to tag SNPs chosen to optimize genomic coverage, this genotyping array contains over 200,000 exon variants that have a higher probability of influencing function of their associated gene products. After cells were trypsinized and pelleted, samples were processed on a spin column using the QIAamp DNA Mini Kit (Qiagen, Valencia, CA) with a final PCR-inhibitor-removal step using the OneStep PCR Inhibitor Removal kit (Zymo Research, Irvine, CA.)

**Fig 2:**
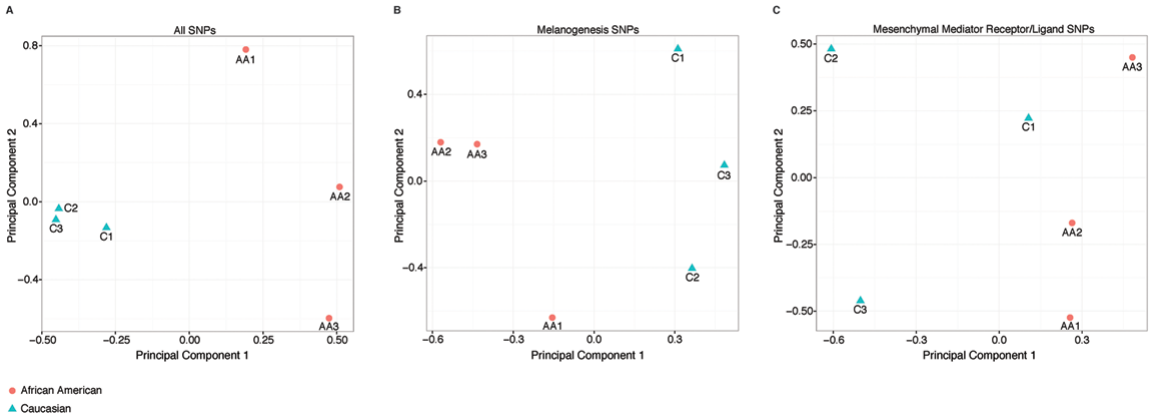
Genetic heterogeneity in melanogenesis-pathway genes underlies variable melanin production. Three Caucasian (C1-3) and three African American (AA2-3) melanocyte lineages were genotyped for >500,000 single nucleotide polymorphisms (SNPs). Principal component analysis (PCA) was performed based on a) the entire genetic profile and in sub-analyses limited b) to pigmentation genes alone or c) genes corresponding to receptors / ligands for the mediators identified in the fibroblast conditioned medium. PCA summarizes genetic variability present across all SNPs by transforming the data into a smaller set of uncorrelated variables called principal components. The first principal component accounts for the highest proportion of the total variability, with each subsequent component accounting for the next highest amount of variability. In these biplots (a, b, and c) of the first two principal components, each point represents an individual sample; points near each other have more similar genetic profiles than those that are farther apart. a) PCA based on all SNPs on the genotyping array demonstrates overall genetic similarity between Caucasian samples compared to African American samples. b) PCA based on SNPs in 102 melanogenesis-pathway genes demonstrates considerably more genetic variability across samples, in accordance with variable melanin production even between samples from individuals of the same race. c) PCA based on 433 SNPs in 22 genes for receptors for the 13 identified fibroblast-secreted cytokines demonstrated high genetic variability between samples and did not cluster samples according to race. Melanocyte lineage responsiveness to fibroblast-conditioned medium could be explained by loss of function in one or more of the receptors to the mediators in the fibroblast derived conditioned medium.

**Fig 3:**
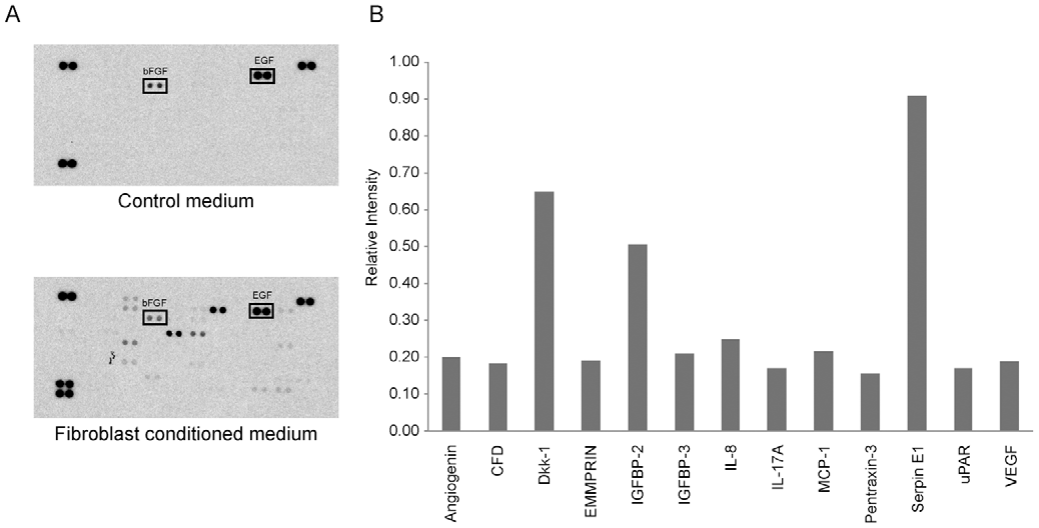
Dermal fibroblast secretome analysis. In order to determine which fibroblast-derived mediators might be responsible for the melanocyte responses, we performed a proteome analysis. Densitometry (b) of the protein spots on the antibody array film targeting 102 human cytokines (a) indicated 13 proteins that were unique to the dermal fibroblast-conditioned medium. The growth factors EGF and bFGF are components of the fibroblast medium and are unlikely to be responsible for the observed paracrine effects by the conditioned medium on the melanocytes since the control cells were exposed to fresh fibroblast medium.

To assess genetic similarity between melanocyte samples, we performed principal component analysis [24,25] (PCA) of polymorphisms in all genes (Fig 2a), in 102 pigmentation genes (Fig 2b), and in 22 genes for receptors /ligands for 13 proteins (Fig 2c) identified in the fibroblast-conditioned medium (Fig 3). The pigmentation genes were those included in the Kyoto Encyclopedia of Genes and Genomes [26] melanogenesis pathway. We specifically examined melanocyte genotypes at the NCKX5-111 and OCA2/P loci that have been associated with increased melanin production [27]. Genotype data were analyzed using PLINK v.1.9 [28] and visualized using R v.3.0.2.

### Human dermal fibroblasts

Primary human adult dermal fibroblasts from a single female donor were purchased (Life Technologies, Grand Island, NY). Cryopreserved cells were thawed and expanded in Medium 106 containing low serum growth supplement (LSGS) and penicillin-streptomycin per company recommendations. Cells were used at passages 4–7 for experiments. Since our interest was to determine differential responses by melanocytes to mesenchymal responses, we restricted our studies to a single fibroblast isolate.

For the conditioned medium, fibroblasts were seeded at 5x10^3^ cells/cm^2^ in Medium 106 containing low serum growth supplement and penicillin-streptomycin in T150 flasks. At 70–80% confluence, fibroblasts were incubated for an additional 48 hours in the 1:1 fibroblast/melanocyte media M106/M254.

### Proteome profiling

Dermal fibroblast-conditioned medium was analyzed for the presence of 102 cytokines using a Proteome Profiler Human XL Cytokine Array (R&D Systems, Inc., Minneapolis, MN) according to the manufacturer protocol. Conditioned supernatant was incubated overnight with a nitrocellulose membrane spotted with capture antibodies in duplicate. After a series of washes, the membrane was incubated with a cocktail of biotinylated antibodies. Protein-detection antibodies bound to the capture antibody were detected using streptavidin-HRP and chemiluminescent detection reagents; signal was captured on Kodak biomax MR film, which was scanned using a flat bed scanner (Epson Perfection 2400 photo). Images were imported into Photoshop CS3® and mean pixel density values were quantified and expressed as average relative pixel intensity.

### Melanocyte stimulation with fibroblast-derived mediators

Fibroblast-conditioned medium was pooled 1:1 with fresh M106/M254 medium and applied to the melanocytes in 6-well tissue culture plates (seeded at 100,000 cells per well) for proliferation and dendrite assays; fresh M106/M254 medium served as a control for these experiments. For the gene expression studies, melanocytes were seeded into in 6-well plates at 6x10^5^ cells per well.

### Melanocyte proliferation assays

Melanocyte proliferation in response to fibroblast-conditioned medium was determined at 48 hours by trypsinizing and counting cells by trypan blue exclusion using a Vi-cell Viability Analyzer (Beckman Coulter, Miami, FL).

### Melanocyte dendrite quantification

Digital images of melanocytes after a 48 hour exposure to fibroblast-conditioned medium were acquired with a Nikon Digital Sight Camera (DS-Fi1) mounted on a Nikon TE300 inverted light microscope equipped with phase contrast objectives. Images were imported into Adobe Photoshop CS3® for analysis. Blinded evaluators analyzed six 10x fields per condition, totaling over 250 cells per treatment group. Dendrite length and number per cell were counted manually; results are expressed as percent of cells with greater than 2 dendrites, average total dendrite length per cell um), and average number of dendrites per cell.

### Real time quantitative reverse-transcription PCR

Melanocyte RNA was extracted using Trizol Reagent (Invitrogen, Carlsbad, CA) followed by purification with the PureLink RNA Mini Kit (Life Technologies, Grand Island, NY), which includes a DNAse digestion step. Since melanin has been reported to interfere with PCR [29], RNA samples were purified using the OneStep™ PCR Inhibitor Removal Kit (Zymo Research Corporation, Irvine, CA), and cDNA was synthesized using an Omniscript RT kit (Qiagen, Valencia, CA).

Real time PCR was performed using a ViiA^TM^7 instrument (Applied Biosystems, Foster City, CA) with the Quantitect SYBR green PCR kit (Qiagen, Valencia, CA). Primers (Table 1) were designed in our laboratory or based on previously published sequences [30]. A dissociation curve for each primer set ensured amplification of a single specific product. The comparative C_*t*_ method (2^-ΔΔCt^) was used to quantify gene expression levels (), where ΔΔC_*t*_ = ΔC_*t*_(sample)– ΔC_*t*_. For all results, the sample represents melanocytes cultured with dermal fibroblast-conditioned medium and the reference represents control melanocytes cultured with an empty insert. Sample and reference were normalized to the endogenous housekeeping gene, GAPDH; dermal fibroblasts had no significant effect on melanocyte GAPDH cycle thresholds. Data are reported as mRNA fold change.

**Table 1:** Primer sequences used for real-time PCR.

	Forward (5’-3’)	Reverse (5’-3’)
MC1R [30]	ACCTGCACTCACCCATGTACTG;	CACGTTGCTCCCGCTCAC
MITF	CCGTCTCTCACTGGATTGGTG	CGTGAATGTGTGTTCATGCCTGG
TYR	CATTCTTCTCCTCTTGGCAGA	CCGCTATCCCAGTAAGTGGA
TYRP1	GCTTTTCTCACATGGCACAG	GGCTCTTGCAACATTTCCTG
TYRP2	CGACTCTGATTAGTCGGAACTCA	GGTGGTTGTAGTCATCCAAGC
GAPDH	ACGGGAAGCTTGTCATCAAT	TGGACTCCACGACGTACTCA
DKK1	ATGCGTCACGCTATGTGCT	CCCATCCAAGGTGCTATGAT

### Statistical analysis

Values are expressed as a mean ± standard deviation. We tested for differences in melanin production based on NCKX5-111 and OCA2/P genotype using an unpaired two-tailed *t*-test. All other statistical testing was based on mean responses across all samples rather than individual sample responses to experimental conditions. For each assay, we fit a linear regression model with robust standard errors as follows:

E[Ra|S,C] = ß0,a + ß1,a×S + ß2,a×C + ß3,a×S×C

where *E*[*R*_*a*_ *|S*,*C*] is the estimated mean response *R* (e.g., cell number) in assay *a* (e.g., proliferation), *S* is an indicator of the melanocyte sample ID, *C* is a dummy variable indicating experimental group (conditioned medium vs. control), and *ß*_*i*_ are regression coefficients. We then used appropriate post-estimation tests of the regression coefficients to answer the following scientific questions: 1) was there significant variation between the six melanocyte samples at baseline? 2) on average, considering all six samples, did conditioned medium have an effect? 3) if so, did the response to conditioned medium vary across the six melanocyte samples? For each scientific question of interest, statistical inference was based on a partial *F*-test of the relevant coefficient estimate(s): (1) *ß*_1,a_ to test for differences across untreated melanocyte samples, (2) *ß*_2,a_ and *ß*_3,a_ simultaneously to test for an overall effect of conditioned medium, and, if (2) was significant, (3) *ß*_3,a_ alone to test whether the response to conditioned medium varied across melanocyte samples. Regression modeling of gene expression was performed using target-gene C_*t*_ values after subtraction of GAPDH C_*t*_ values. Statistical analyses were performed using Stata v.13.0 (StataCorp, College Station, TX), and *p≤*0.05 was considered significant. All experiments included 4–6 replicates and were repeated at least three times.

## Results

### Melanocyte Characterization

Intracellular melanin content (Fig 1) varied across the melanocyte samples both at baseline (*p*<0.0001) and in response to conditioned medium (*p* = 0.01); extracellular melanin was not detectable. In addition, the magnitude of response to conditioned medium varied significantly according to melanocyte sample (*p* = 0.03).

To assess genetic differences underlying this heterogeneous phenotypic response, we performed principal component analysis (PCA) of DNA single nucleotide polymorphisms (SNPs) using >500,000 SNPs spanning the whole genome (S1 Table; Fig 2a) as well as a subset of 1,887 SNPs in melanogenesis-pathway genes (Fig 2b). Regarding genome-wide SNPs, melanocytes from Caucasian individuals (C1-3) clustered together; however, the Hispanic sample (C1) was slightly distinct from the non-Hispanic Caucasian samples (C2 and C3). African American samples (AA 1–3) separated from Caucasians (C1-3), and were considerably disparate from each other suggesting wide genetic variability (Fig 2a). Focus on melanogenesis-specific genes (Fig 2b) identified greater genetic variability between the Caucasian cell lines (C1-3) and tighter clustering of two of the AA cell lines (A2 and A3). Since melanin production has previously been shown to be increased in melanocytes with SNPs at the NCKX5-111 and OCA2/P loci [27], we specifically tested for differences in baseline melanin production between samples either hetero- or homozygous for the variant allele compared to those homozygous for the common allele. Consistent with a previous report [27], average melanin production was greater (p = 0.06) among melanocytes with at least one variant allele at the NCKX5-111 locus (A1 and A2) compared to those with no variant alleles (C1-3 and A3). We did not detect an association between OCA2/P genotype and melanin content (*p* = 0.63) in our samples.

### Dermal fibroblasts alter melanocyte proliferation and dendricity

**Fig 4:**
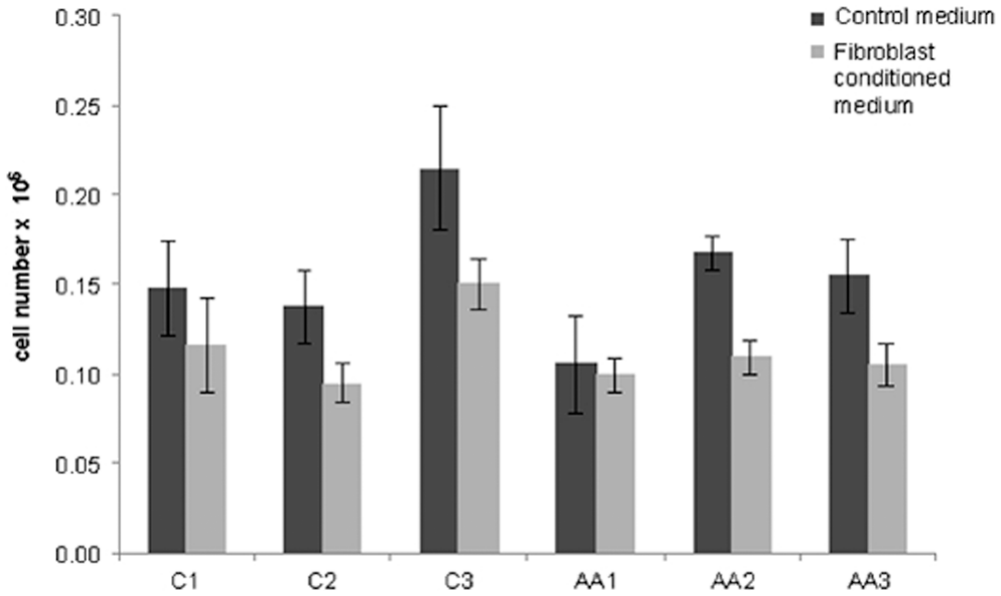
Dermal-fibroblast-derived paracrine signaling inhibits melanocyte proliferation. Melanocyte proliferation varied significantly across melanocyte lineages at baseline (*p* = 0.0002). Dermal fibroblast-conditioned medium significantly inhibited melanocyte proliferation at 48 hours (*p*<0.0001), and the degree of inhibition varied significantly between samples (*p* = 0.05). The data in this graph represents a single experiment in which N = 4; the experiment was reproduced three times. Statistical analysis was based on average values across all six melanocyte lineages rather than individual sample responses to fibroblast-conditioned medium (see Methods).

**Fig 5:**
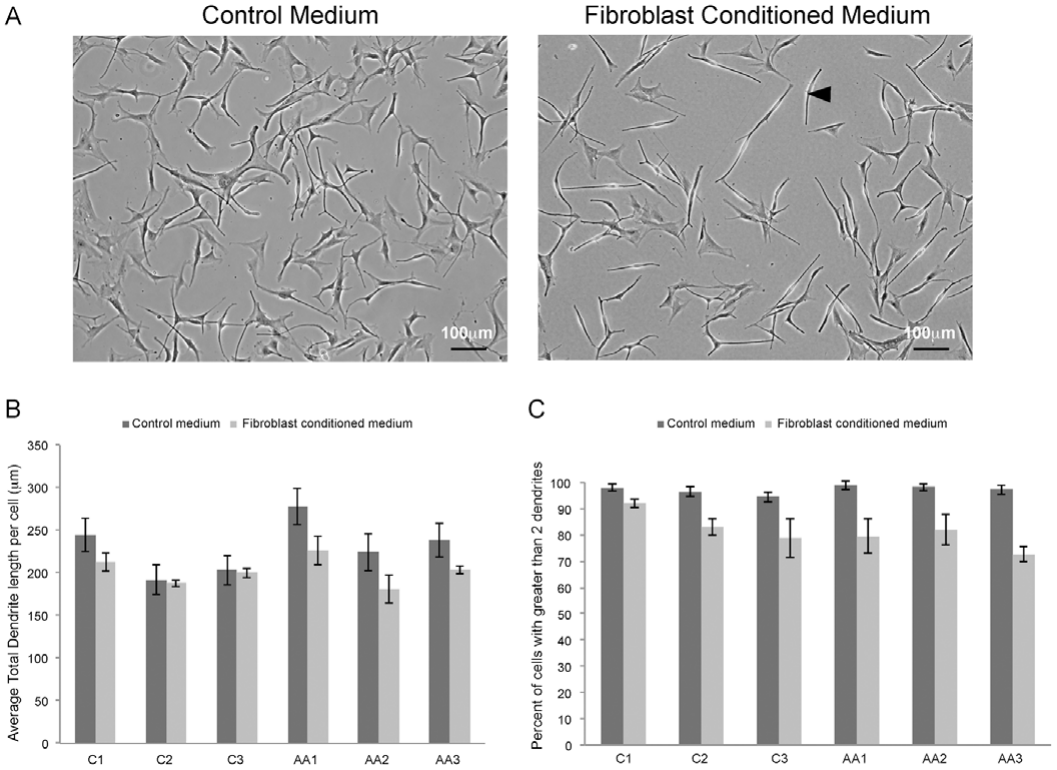
Dermal-fibroblast-derived paracrine signaling reduces melanocyte dendricity. Dendricity was determined by analysis of melanocytes cultured without (a) and with (b) fibroblast conditioned medium. Average dendrite length (c) was significantly different between untreated samples at baseline (*p*<0.0001) and decreased significantly in response to fibroblast-conditioned medium (*p* = 0.005). The fraction of cells with >2 dendrites (d) also varied significantly at baseline (*p* = 0.002) and decreased in response to conditioned medium (p<0.0001). Dendricity in response to conditioned medium was not consistent between samples from individuals of the same race. Statistical analysis was based on average values across all six melanocyte lineages rather than individual sample responses to fibroblast-conditioned medium (see Methods).

Baseline proliferation varied significantly across melanocyte samples (*p* = 0.0002; Fig 4). Fibroblast-conditioned medium significantly decreased melanocyte proliferation (*p*<0.0001); the magnitude of the decrease differed significantly between samples (*p* = 0.05). Similarly, dendricity decreased in response to fibroblast-conditioned medium across samples (Fig 5a). Average dendrite length (Fig 5b) varied significantly across untreated samples (*p*<0.0001) and decreased significantly in response to fibroblast-conditioned medium (*p* = 0.005); the magnitude of the decrease varied based on sample (*p* = 0.04). The proportion of multipolar cells (>2 dendrites) also varied significantly at baseline (Fig 5c; *p* = 0.002) and decreased in response to conditioned medium, with the magnitude of the reduction dependent on the melanocyte sample (*p*<0.0001). These data have clinical implications, since dendrites are critical for transfer of melanosomes to keratinocytes and dendrite number determines melanocyte activity in terms of pigment production [13,31,32]. We have previously published evidence that conditioned medium modulates endothelial cell and fibroblast in vitro responses. [33,34] This coupled with the observation that melanocyte isolate phenotypic responses differed from one another and depending on the read-out suggests that the responses are not artifacts of conditioned medium.

Our results confirm that melanocyte responses to injury cannot be generalized by race, as melanocytes from individuals of the same self-reported race showed considerable phenotypic variation.

### Dermal fibroblasts alter melanocyte gene expression

**Fig 6:**
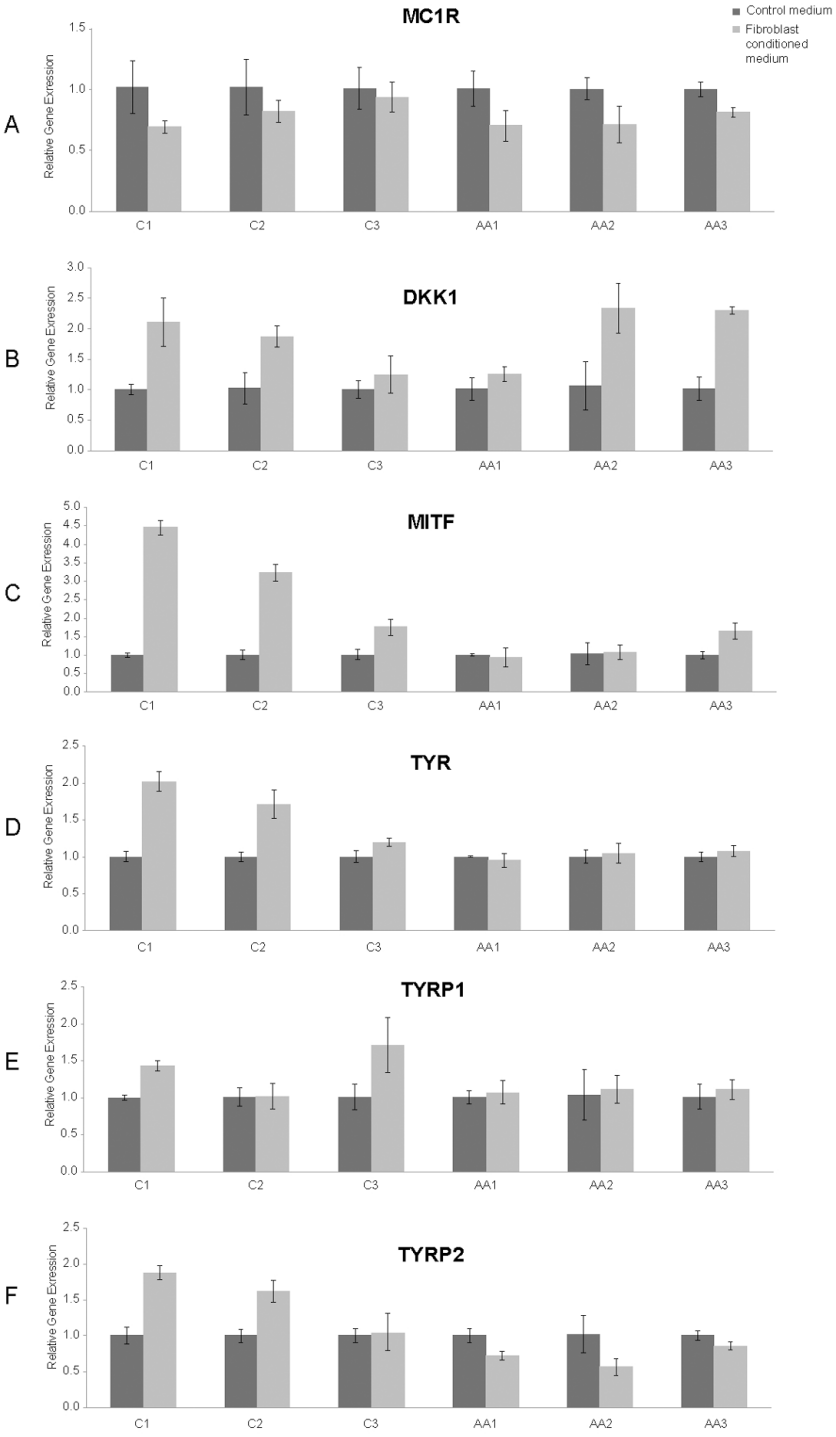
Dermal fibroblast derived paracrine signaling regulates expression of genes involved in melanin biosynthesis. Baseline expression of 6 genes involved in melanogenesis varied significantly between the melanocyte samples (*p*<0.01 for all genes); conditioned medium significantly affected gene expression (*p*<0.001 for all genes). The degree of response to conditioned medium was significantly different between melanocyte samples for MITF (*p*<0.0001), TYR (*p*<0.0001), TYRP1 (*p* = 0.02), and TYRP2 (*p<*0.0001) and was nearly significant for DKK1 (*p* = 0.07), but not for MC1R (*p* = 0.22). Statistical analysis was based on average values across all six melanocyte lineages rather than individual sample responses to fibroblast-conditioned medium (see Methods).

Gene expression (Fig 6) by the different melanocyte lineages confirmed that race alone does not explain heterogeneous responses. Baseline expression for each of the 6 genes varied significantly across all six melanocyte lineages (*p*<0.01 for all genes). Conditioned medium significantly affected expression of all genes (*p*<0.001 for all genes). The magnitude of the response to conditioned medium varied significantly across melanocyte samples for MITF (*p*<0.0001), TYR (*p*<0.0001), TYRP1 (*p* = 0.02), and TYRP2 (*p<*0.0001) but not for MC1R (*p* = 0.22) or DKK1 (*p* = 0.07). The closest correlation between race and gene expression occurred in two well-recognized determinants of pigment production, tyrosinase (TYR) and tyrosine-related protein 2 (TYRP2 or DCT). Fibroblast-conditioned medium increased TYR expression in all Caucasian (C1-3) melanocytes, but not in the African American (AA1-3) cells (Fig 6d). TYRP2 expression decreased in response to the fibroblast-conditioned medium in all of the African American (AA1-3) melanocytes, but not in the Caucasian cells (C1-3; Fig 6f). Since tyrosinase is the enzyme responsible for the first step in melanin production and tyrosine-related protein 2 is only associated with eumelanin melanosomes and not pheomelanin melanosomes [23], these results offer a potential pathophysiology for abnormal pigmentation in healed wounds and scars following partial thickness injury.

### Dermal fibroblast-derived soluble mediator characterization

Analysis of the dermal fibroblast secretory protein profile in the conditioned medium identified 13 soluble paracrine mediators unique to dermal fibroblast-conditioned medium compared to control medium (S2 Table; Fig 3): angiogenin, CFD, DKK1, EMMPRIN, IGFBP-2, IGFBP-3, IL-8, IL-17A, MCP-1, Pentraxin-3, Serpin E1, uPAR, and VEGF. These proteins are potential targets for therapeutic modulation. Epidermal growth factor (EGF) and basic fibroblast growth factor (bFGF) were detected in both the control and experimental medium without differences in concentration, confirming that they are components of the base medium.

Hypothesizing that genetic variation in receptors / ligands for the 13 identified fibroblast-secreted proteins might explain the variable melanocyte responses to fibroblast-conditioned medium, we performed principal component analysis (PCA) of 433 SNPs in 22 genes for receptors / ligands for the 13 soluble fibroblast-secreted mediators (Fig 2c). PCA based on receptor genotype showed considerable genetic variation between samples and did not cluster the samples by race. The functions of the ligands, applicability to wound repair and SNP classification are included in a Supplemental Table. Receptor genotype may explain heterogeneous melanocyte responses to soluble fibroblast-secreted mediators.

## Discussion

Whereas keratinocyte-derived paracrine factors strongly influence melanocyte proliferation, migration, and differentiation [32], growing evidence suggests that dermal fibroblasts also modulate melanocyte behavior [16,35–39]. This epidermal-mesenchymal interaction may be especially relevant to slowly healing wounds in which an immature basement membrane may allow passage of soluble paracrine mediators between the dermal and epidermal compartments. Unpredictable pigmentation in healed wounds depends on the baseline skin tone, depth of wound, and healing time. Wounds that take longer to heal often have abnormal pigmentation [3,7]. Therefore, we hypothesized that dermal fibroblasts regulate melanocyte responses to cutaneous injury.

Our data underscore the heterogeneity of melanocytes [27,40,41]. Together with published observations that fibroblast-melanocyte interactions in palmo-plantar skin are unique [39], our results reinforce the importance of knowing the cellular tissue source and genetic profile of cell isolates. In our study, reported race correlated poorly with melanocyte phenotypic responses, including melanin production, proliferation and dendrite formation. Interestingly, principal component analysis of SNP genotype data for SNPs in all genes (Fig 2a) clustered the Caucasian melanocyte lineages more closely than when the analysis was restricted to genes involved in either melanogenesis (Fig 2b) or receptors for fibroblast-secreted proteins (Fig 2c). Whereas the C1 isolate, derived from a Hispanic donor is slightly disparate from the other two Caucasian samples, none of our subsequent analyses suggest differential responses by this cell line.

The uniformly increased tyrosinase expression in melanocytes isolated from Caucasians, but not African Americans, suggests that this enzyme, which regulates the rate-limiting step in conversion of tyrosine to melanin [23], is a key determinant of melanocyte responsiveness after injury. Our data are corroborated by a recent publication [42] that shows that melanocytes with homogeneous wild type TYR alleles had higher TYR protein levels and enzyme activity than other genotypes and that melanocytes that were homozygous for the variant TYR allele produced significantly less TYR protein and displayed altered trafficking and glycosylation. Likewise, our observation that African American melanocytes have reduced tyrosinase related peptide 2 (or dopachrome tautomerase) expression in response to fibroblasts is novel. Since TYRP2 is present in eumelanosomes and not pheomelanosomes [23], individuals with darker skin would be more likely affected by reduced TYRP2 expression, suggesting a potential etiology for hypopigmentation in scars in individuals of color. These in vitro data support the need for future genetic association studies to correlate TYRP2 expression with human scarring.

Several of the 13 profibrotic and proinflammatory mediators in the fibroblast-conditioned medium, including IGFBP 2 and 3, IL8, IL17, MCP1 and VEGF, would be anticipated to be involved in wound repair processes (Table 2); whether these mediators directly regulate melanocyte responses must be determined. Of the differentially expressed soluble mediators in the conditioned medium, fibroblast-derived dickkopf-1 (DKK1) may be most relevant since it has been associated with hypopigmentation in palmo-plantar skin [43] and epidermal responses to injury, especially since the palmar and plantar surfaces rarely scar [44]. Our data indicate that in response to fibroblast-conditioned medium, the AA1 melanocyte lineage had minimally increased DKK1 and the most dramatic increase in melanin production, supporting a role of DKK1 in modulating melanogenesis. Interestingly, in the melanogenesis specific PCA, this AA1 isolate did not cluster with the other AA samples, suggesting a potential explanation for the variable melanin production by the AA1 melanocytes compared to the samples isolated from individuals identified as the same race. With the small number of melanocyte samples (6) relative to the large number of candidate SNPs on the genearray, genome wide association testing to correlate specific SNPs with any phenotypic or genetic responses is not possible.

**Table 2:** 

Fibroblast-Derived Mediator	Potential Cutaneous Wound Repair Roles
Angiogenin	Strongly angiogenic ribonuclease that binds to actin on endothelial cells; internalized by endocytosis and translocated to the nucleus.
Complement Factor D(Adipsin)	Serine protease component of the alternative complement pathway, secreted by adipocytes suggesting a role for adipose tissue mediated immune responses.
Dkk-1 (Dickkopf-related protein 1)	Secreted protein that regulates morphogenesis by inhibiting Wnt signaling and inhibits melanocyte melanin production in palmar skin
EMMPRIN Extracellular matrix metalloproteinase inducer (CD 147)	Cell surface glycoprotein known for ability to induce matrix metalloproteinase production and to promote myofibroblast differentiation and cutaneous neoplastic metastasis.
IL-8(Neutrophil-Activating Peptide 1)	Chemokine, secreted by multiple cell types in response to inflammation and injury, that attracts neutrophils, basophils, and T-cells, but not monocytes and is involved in neutrophil activation.
IL-17A (Cytotoxic T-Lymphocyte-Associated Antigen 8)	Proinflammatory cytokine that induces proinflammatory cytokines including IL6 and cyclooxygenase-2 and nitric oxide. Enhances ICAM1 expression in fibroblasts and may promote angiogenesis
IGFBP2, 3 (Insulin-Like Growth Factor Binding Protein)	Circulating proteins with anti-proliferative effects that alter IGF binding to cell surface receptors and inhibit IGF-mediated growth processes.
MCP-1 (Monocyte Chemotactic Protein 1)	Chemokine secreted by monocytes/ macrophages, lymphocytes, endothelial cells, fibroblasts, and non pigmented melanocytes; responsible for recruiting mononuclear cells into inflammation sites and stimulates angiogenesis by increasing endothelial cell proliferation and migration
Pentraxin-3 (TNF Alpha-Induced Protein 5)	Secreted innate immune pattern recognition molecule produced by inflammatory cells and involved in opsonization and complement cascade regulation with deficiencies associated with increased fibrin accumulation, collagen deposition, and epithelial hyperplasia suggesting a role in wound matrix remodeling
Serpin E1 /PAI-1 (Plasminogen Activator Inhibitor Type 1)	Serine protease inhibitor of plasminogen activator, regulating fibrinolysis and as such extracellular remodeling in wounds and tumors
uPAR (Urokinase—Plasminogen Activator Receptor)	Soluble and membrane bound receptor for urokinase plasminogen activator, involved in plasmin production and implicated in extracellular degradation important for tissue remodeling and possibly melanocyte migration
VEGFA(Vascular Endothelial Growth Factor A)	Strongly angiogenic growth factor that promotes endothelial cell proliferation, migration, angiogenesis, vasculogenesis and capillary permeabilization and is implicated in wound healing and tumor growth

Since our results indicates that self-identified race alone does not reliably predict either melanocyte phenotype or genotype, knowledge of patient race is insufficient to predict pigmentation outcomes after cutaneous injury. Our study supports the need for genetic association studies to identify specific genetic variants responsible for post-injury pigmentation changes. The phenotypic variability by melanocytes in response to fibroblast-conditioned medium, coupled with genotypic variation in the PCA focused on SNPs in receptor / ligand genes for soluble fibroblast-secreted mediators (Fig 2c, Supplemental Table), suggests that candidate-gene association studies are warranted to determine whether any of the receptors / ligands for the fibroblast derived mediators (Table 2) correlate with pigmentation changes after injury.

In summary, our data confirm that dermal fibroblast-derived soluble factors modulate melanocyte responses. The heterogeneous melanocyte phenotypic and genetic responses suggest that race and ethnicity alone do not account for phenotype and that genetic association studies are necessary to understand melanocyte responses to injury and to correlate those responses to patient outcomes.

## Supporting Information

S1 Table SNP analysis of the 6 melanocyte cell lines was performed as part of a genome wide association study. The complete results are included as a supplemental dataset that can be accessed using PLINK v.1.9 and R v.3.0.2.
(ZIP)

S2 Table SNP analysis was performed in the melanocytes to determine whether they had mutations in receptors or ligands for proteins identified in the proteome profiler analysis. This table summarizes the SNPs that are included in Illumina Bead Chip.
(XLSX)
